# Pretreatment peripheral neutrophils, lymphocytes and monocytes predict long-term survival in hepatocellular carcinoma

**DOI:** 10.1186/s12885-020-07105-8

**Published:** 2020-09-29

**Authors:** Young Mi Hong, Ki Tae Yoon, Tae Ho Hwang, Mong Cho

**Affiliations:** 1 Liver center, Pusan National University Yangsan Hospital, Department of Internal Medicine, Pusan National University School of Medicine, 20 Geumo-ro, Yangsan, Gyeongnam 50612 Republic of Korea; 2 Department of Pharmacology, Pusan National University School of Medicine, Yangsan, Republic of Korea

**Keywords:** Hepatocellular carcinoma, Immune cells, Prognosis

## Abstract

**Background:**

Hepatocellular carcinoma (HCC) is an inflammation-related cancer, where nonresolving inflammation contributes to its development and progression. Peripheral inflammatory cells have been shown to be associated with the prognosis of various types of cancer. The present study investigated the utility of pretreatment peripheral inflammatory cells in the prognosis of patients with HCC.

**Methods:**

We retrospectively analyzed data regarding peripheral inflammatory cell, and patient and tumor characteristics from patients with HCC who were diagnosed between November 2008 and March 2018. Baseline data, including peripheral inflammatory cell counts, were recorded before treatment. The relationships between overall survival (OS) and study variables were assessed.

**Results:**

A total of 1681 patients who were diagnosed with HCC were included. In univariate and multivariate analyses, individual neutrophil, lymphocyte and monocyte cell counts were found as independent indicators of poor OS. High neutrophil (≥3100 × 10^6^/L) and, monocyte (≥470 × 10^6^/L) counts and low lymphocyte counts (< 1640 × 10^6^/L) significantly associated with reduced OS (*p* < 0.05). Neutrophil and, monocyte cell counts rose and lymphocyte counts decreased in association with advancing the Barcelona Clinic Liver Cancer stage (*P* < 0.001).

**Conclusions:**

Pretreatment peripheral neutrophils, lymphocytes, and monocytes are independently associated with outcomes of patients with HCC. These cells provides a noninvasive, low-cost, easy, and reproducible biomarker that can be used in routine clinical practice to predict the prognosis of patients with HCC.

## Background

Hepatocellular carcinoma (HCC) is one of the most common malignancy worldwide and the fourth most common causes of cancer-related deaths [1]. HCC is an aggressive tumor that frequently occurs in the setting of chronic liver disease and cirrhosis. It is typically diagnosed late, and despite recent treatment, the 5-year survival rate of patients with HCC remains low compared with other cancers [2, 3].

The available therapeutic options for HCC are determined by the complex interaction of tumor stage and extent of underlying liver disease. Although various systems have been proposed for staging and predicting the prognosis of HCC, there is no worldwide consensus as to which staging system is best predicts the survival of patients with HCC [4–10]. In addition, the application of these staging systems can be cumbersome in daily clinical practice. Because there is a lack of validated prognostic biomarkers in HCC, the identification of simple and easily applicable prognostic predictive factors for HCC, such as serum biomarkers, is needed, and reliable prognosis predictions will facilitate the development of more efficacious therapeutic strategies for HCC.

Molecular aberrations in tumor cells are oncogenic drivers in HCC, and interplay with inflammatory cells within the tumor microenvironment may also be key factor for tumor progression [11–16]. Inflammation is also recognized to play an important role in cancer development and mounting evidence has established that excessive systemic or local inflammation facilitates tumor progression [17, 18]. Various inflammatory markers have been suggested as a useful prognostic markers in patients with various types of cancer [19–23]. Previous reports have revealed that the presence of tumor-infiltrating immune cells is associated with tumor progression and clinical response to treatment [24–26]. In addition, these immune cells conduct their functions in the tumor microenvironment, and in the peripheral blood to promote metastasis [27, 28]. In line with these facts, the quantification of peripheral blood inflammatory cells such as neutrophils, lymphocytes, monocytes, and platelets, as well as their ratios, have been identified and validated as novel biomarkers with prognostic significance in several cancers [29–34]. Therefore, we evaluated the prognostic values of pretreatment peripheral inflammatory cells (neutrophils, lymphocytes, monocytes, and platelets) in a retrospective cohort of patients with HCC.

## Methods

### Study populations

We retrospectively analyzed patients with HCC who were newly diagnosed between November 2008 and March 2018. Data on patients’ demographic and clinical characteristics, laboratory results, and imaging findings were collected retrospectively.

The diagnosis of HCC was based on histopathologic or radiologic findings (contrast wash-in during the arterial phase followed by contrast wash-out during the venous or delayed phases) according to guidelines proposed by the Korea Liver Study Group [35].

Patients who had an active infection or inflammatory disease and had received any medication that could affect pretreatment blood tests such as granulocyte-colony stimulating factor (G-CSF) or high-dose steroids were excluded from our study. Patients for whom data regarding the above-mentioned clinical, laboratory, and imaging findings were unavailable were also excluded. All pretreatment routine blood cell examinations that were performed within 2 weeks before treatment were used for the current analysis. Overall survival (OS) was calculated from the date of HCC diagnosis to the date of death or last follow-up. OS was compared for between patients with high and low neutrophils, lymphocytes, and monocytes (dichotomized by median values).

### Statistical analysis

The categorical data are reported as the number or percentage of observations while continuous variables are presented as mean ± standard deviations of the values. The differences between categorical variables were assessed by a chi-square test. Differences between groups of continuous variables that were non-parametrically distributed were assessed using Kruskal–Wallis tests. Univariate analysis of variables was performed by using univariate cox regression analysis. All variables selected on univariate analysis (*p* < 0.05) were included in the multivariate analysis. Multivariate prognostic analyses were performed by using the Cox proportional hazards regression model. A Kaplan–Meier survival analysis with a log-rank test was performed to compare the OS of patients in different the groups. *P* values < 0.05 are considered statistically significant. All statistical procedures were performed using SPSS Windows (version 21; SPSS Inc., Chicago, IL, USA).

## Results

### Patient characteristics and clinical outcomes

A total of 1681 patients with HCC were included in our study. The baseline characteristics of the patients are shown in Table 1. Our study included 1335 (79.4%) males and 346 (20.6%) females. The patients ranged in age from 27 to 88 years, and the median age was 60 years. Hepatitis B, hepatitis C, and alcoholic liver disease were diagnosed in 1039 (61.8%), 279 (16.6%), and 184 (11.0%) patients, respectively. The frequency of patients with underlying liver cirrhosis was 85.1%.

**Table 1:** Patients characteristics

Variables	No.(%)/median (range)
Age	60 (27–88)
Gender	
Male	1335 (79.4%)
Female	346 (20.6%)
Etiology	
HBV	1039 (61.8%)
HCV	279 (16.6%)
Alcohol	184 (11.0%)
Others	179 (10.6%)
Cirrhosis	1431 (85.1%)
Tumor characteristics	
Number (single)	1151 (68.5%)
Size, cm	3.2 (1–20)
Vascular invasion	313 (18.6%)
Extrahepatic metastases	136 (8.1%)
BCLC stage	
Stage A	764 (45.4%)
Stage B	495 (29.5%)
Stage C	363 (21.6%)
Stage D	59 (3.5%)
Child-Pugh	
Child-Pugh A	1454 (86.5%)
Child-Pugh B	202 (12.0%)
Child-Pugh C	25 (1.5%)
Peripheral cell counts, 10^6^/L	
Neutrophil	3100 (630–17,500)
Lymphocyte	1640 (270–7250)
Monocyte	470 (60–2030)
Platelet	145,000 (17,000–685,000)
Primary treatment	
Resection	407 (24.2%)
Liver transplantation	51 (3.0%)
RFA	138 (8.2%)
TACE	710 (42.2%)
Sorafenib	179 (10.7%)
Others	14 (0.8%)
Radiotherapy	12 (0.7%)
Supportive care	171 (10.2%)

Abbreviations: *BCLC* Barcelona clinic liver cancer; *HBV* Hepatitis B virus; *HCV* Hepatitis C virus; *RFA* Radiofrequency ablation; *TACE* Transarterial checmoembolization

The median tumor size was 3.2 cm, and 1151 (68.5%) patients had single tumor. Vascular invasion was observed in 313 (18.6%) patients, and 136 (8.1%) patients had extrahepatic metastasis. Seven hundred sixty-four (45.4%), 495 (29.5%), 363 (21.6%) and 59 (3.5%) patients were classified as Barcelona Clinic Liver Cancer (BCLC) stage A, B, C, and D, respectively. At the time of diagnosis, 1454 (86.5%), 202 (12.0%), and 25 (1.5%) of the patients were classified as Child–Pugh class A, B, or C, respectively.

Surgical resection as primary treatment was performed in 407 (24.2%) patients, liver transplantation was performed in 51 (3.0%), locoregional treatments were performed in 848 (50.4%) and sorafenib was administered in 171 (10.7%) patients. Until the data cut-off day (2018.10.31.), 812 (48.3%) patients remained alive. The median OS period was 59 months and the 5-year OS rates was 38.4%.

### Prognostic predicting factors of pretreatment immune cell counts

The prognostic value of various clinical variables for identifying predictive factors were evaluated. The univariate analysis showed that age, sex, etiology (HBV), alcohol, underlying liver cirrhosis, tumor number (multiple), tumor size ≥3.2 cm, vascular invasion, extrahepatic metastasis, α-Fetoprotein (AFP) ≥ 100 ng/mL, Protein induced by vitamin K absence or antagonist-II (PIVKA) ≥100 mAU/L, albumin, total bilirubin, prothrombin time, lactate dehydrogenase (LDH), and neutrophil, lymphocyte, monocyte counts were associated with OS in patients with HCC (*P* < 0.05, Table 2). In the multivariate Cox regression analysis, tumor number (multiple) (hazard ratio [HR] 1.262; 95% confidence interval [95% CI] 1.031–1.545, *P* = 0.024), tumor size ≥3.2 cm (HR 1.315; 95% CI 1.018–1.698, *P* = 0.036), vascular invasion (HR 4.335; 95% CI 3.326–5.649, *P* < 0.001), extrahepatic metastasis (HR 2.625; 95% CI 1.925–3.578, *P* < 0.001), AFP ≥ 100 ng/mL (HR 1.501; 95% CI 1.212–1.859, *P* < 0.001), PIVKA ≥100 mAU/L (HR 1.592; 95% CI 1.212–2.032, *P* < 0.001), albumin (HR 2.082; 95% CI 1.654–2.621, *P* < 0.001), LDH (HR 1.253; 95% CI 1.020–1.541, *P* = 0.032), neutrophil counts (HR 1.348; 95% CI 1.089–1.669, *P* = 0.006), lymphocyte counts (HR 0.685; 95% CI 0.559–0.841, *P* < 0.001) and monocyte counts (HR 1.338; 95% CI 1.083–1.645, *P* = 0.007) were independent prognostic factors for OS (Table 2).

**Table 2:** Predictors of survival: Univariate and Multivariate survival analyses

	Univariate		Multivariate	
	*P* value	HR (95% CI)	*P value*	HR (95% CI)
Age (years) ≥ 60	0.002	1.240 (1.085–1.418)	0.153	
Gender (male)	0.012	1.246 (1.049–1.480)	0.675	
Etiology (HBV)	< 0.001	0.763 (0.666–0.873)	0.563	
Alcohol	0.044	1.147 (1.003–1.310)	0.483	
Smoking	0.155	1.102 (0.964–1.259)		
Cirrhosis	0.001	1.400 (1.141–1.719)	0.760	
Tumor number (multiple)	< 0.001	1.610 (1.404–1.845)	0.024	1.262 (1.031–1.545)
Tumor size ≥3.2 (cm)	< 0.001	2.997 (2.591–3.466)	0.036	1.315 (1.018–1.698)
Vascular invasion	< 0.001	7.089 (6.098–8.240)	< 0.001	4.335 (3.326–5.649)
Extrahepatic metastases	< 0.001	5.123 (4.228–6.219)	< 0.001	2.625 (1.925–3.578)
AFP ≥100 (ng/L)^a^	< 0.001	2.238 (1.946–2.575)	< 0.001	1.501 (1.212–1.859)
PIVKA-II ≥100 (mAU/L)^b^	< 0.001	3.200 (2.628–3.897)	< 0.001	1.592 (1.247–2.032)
Albumin < 4 (g/dL)	< 0.001	0.400 (0.349–0.458)	< 0.001	2.082 (1.654–2.621)
Total bilirubin ≥0.9 (mg/dL)	< 0.001	1.797 (1.560–2.018)	0.080	
PT time ≥ 12.8 (sec)	0.007	1.218 (1.169–1.421)	0.108	
LDH ≥439 (IU/L)^**c**^	< 0.001	1.964 (1.716–2.248)	0.032	1.253 (1.020–1.541)
Neutrophil ≥3100 (×10^6^/L)	< 0.001	1.664 (1.454–1.903)	0.006	1.348 (1.089–1.669)
Lymphocyte ≥1640 (×10^6^/L)	< 0.001	0.622 (0.543–0.711)	< 0.001	0.685 (0.559–0.841)
Monocyte ≥470 (×10^6^/L)	< 0.001	1.656 (1.147–1.896)	0.007	1.338 (1.083–1.645)
Platelet ≥145,000 (×10^6^/L)	0.113	1.105 (0.973–1.275)		

Abbreviations: *AFP* α-Fetoprotein; *HBV* Hepatitis B virus; *CI* Confidence interval; *HR* Hazard ratio; *LDH* Lactate dehydrogenase; *PIVKA-II* Protein induced by vitamin K absence or antagonist-II; *PT* Pothrombin time

^a^*n* = 1568, ^b^*n* = 1089, ^c^*n* = 1622

### Relation between pretreatment immune cell counts and overall survival

**Fig. 1:**
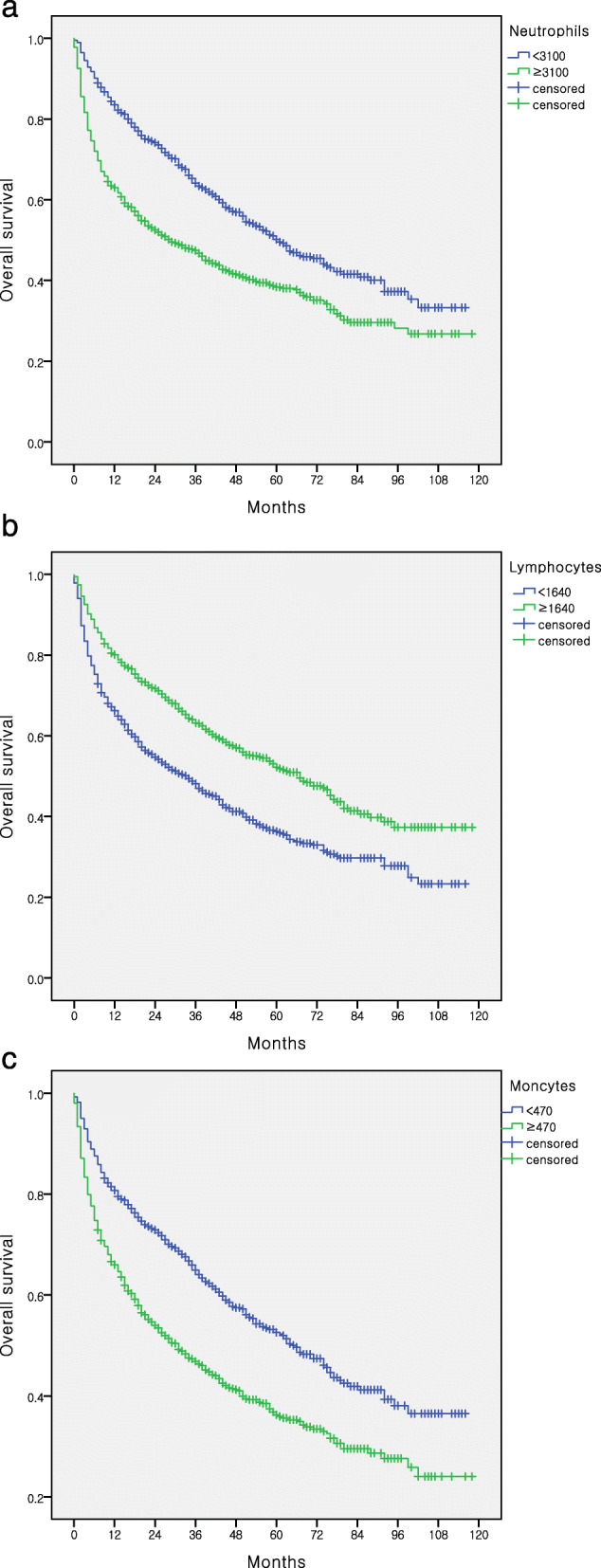
Immune cells associations with survival

**Fig. 2:**
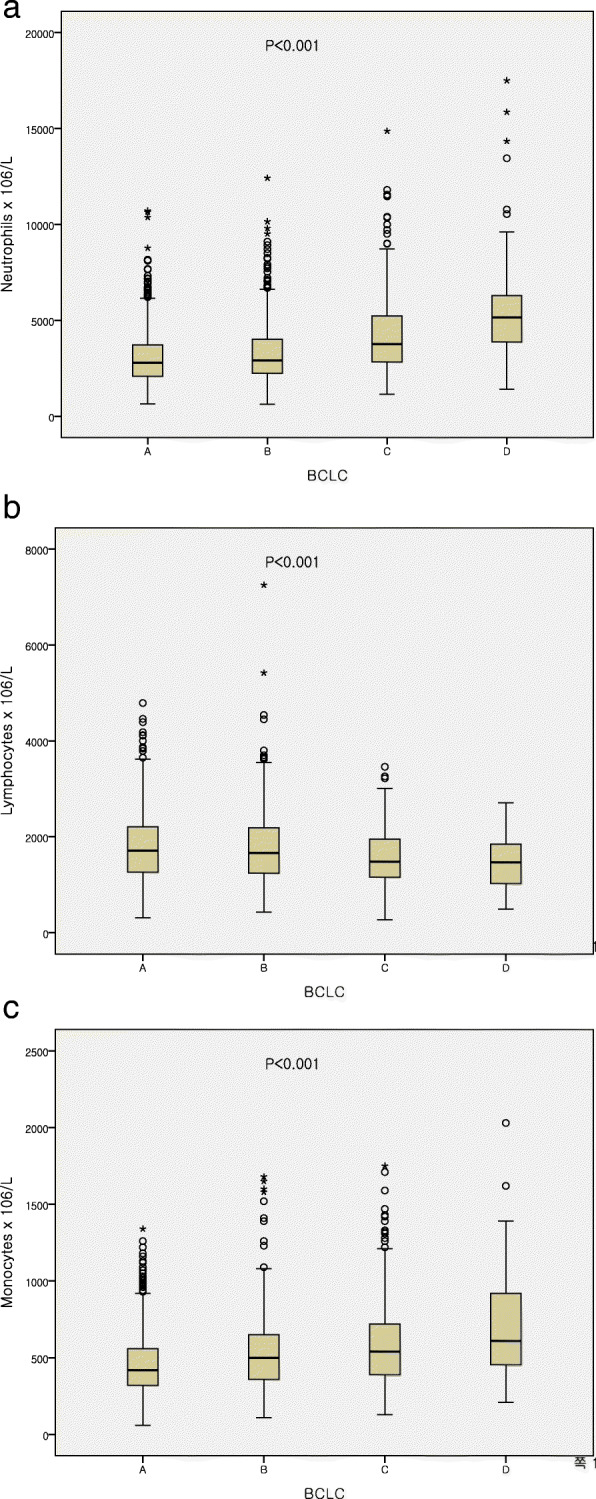
Relationship between pretreatment immune cell counts and BCLC stage

All patients were stratified into high and low subgroups based on high or low neutrophil, lymphocyte and monocyte counts. Kaplan–Meier analyses revealed that patients with higher neutrophil or monocyte counts showed significantly poor prognosis, while those with lower lymphocyte counts had worse OS (Fig. 1a–c). Neutrophil and monocyte counts increased with advanced tumor stage, whereas lymphocyte counts decreased (Fig. 2a-c). We also analyzed the combination score and found that higher neutrophil to lymphocyte ratio (NLR), platelet to lymphocyte ratio (PLR) and lower lymphocyte to monocyte ratio (LMR) had worse OS (data not shown).

### Association of pretreatment immune cell counts with clinical factors

The associations between clinical variables and neutrophil, lymphocyte, and monocyte counts were further analyzed. As shown in Table 3*,* patients with high neutrophils and monocytes were more likely to have larger tumor size (*p* < 0.001)*.* The presence of vascular invasion was associated with high neutrophil and, monocyte counts and with low lymphocyte counts (*p* < 0.001)*.* High neutrophils and monocyte counts were associated with extrahepatic metastasis (*p* < 0.001).

**Table 3:** Associations between neutrophil, lymphocyte, monocyte and clinical factors in HCC

		Neutrophils			Lymphocytes			Monocytes		
**Variables**		**Low (*N*** **= 838)**	**High (*N*** **= 843)**	***P value***	**Low (*N*** **= 836)**	**High (*N*** **= 845)**	***P value***	**Low (*N*** **= 821)**	**High (*N*** **= 860)**	***P value***
Age (years)	< 60	411 (49%)	412 (48.9%)	0.149	389 (46.5%)	434 (51.4%)	0.051	391 (47.6%)	432 (50.2%)	0.305
	≥ 60	427 (51%)	431 (51.1%)		447 (53.5%)	411 (48.6%)		430 (52.4%)	428 (49.8%)	
Gender	Female	212 (25.3%)	134 (15.9%)	< 0.001	201 (24%)	145 (17.2%)	0.001	247 (30.1%)	99 (11.5%)	< 0.001
	Male	626 (74.7%)	709 (84.1%)		635 (76%)	700 (82.8%)		574 (69.9%)	761 (88.5%)	
Alcohol	No	418 (49.9%)	397 (47.1%)	0.262	463 (55.4%)	352 (41.7%)	< 0.001	468 (57%)	347 (40.3%)	< 0.001
	Yes	420 (50.1%)	446 (52.9%)		373 (44.6%)	493 (58.3%)		353 (43%)	513 (59.7%)	
Smoking	No	499 (59.5%)	442 (52.4%)	0.004	540 (64.6%)	401 (47.5%)	< 0.001	534 (65%)	407 (47.3%)	< 0.001
	Yes	339 (40.5%)	401 (47.6%)		296 (65.4%)	444 (52.5%)		287 (35%)	453 (52.7%)	
Etiology (HBV)	No	326 (38.9%)	316 (37.5%)	0.581	311 (37.2%)	331 (39.2%)	0.422	256 (31.2%)	386 (44.9%)	< 0.001
	Yes	512 (61.1%)	527 (62.5%)		525 (62.8%)	514 (60.8%)		565 (68.8%)	474 (55.1%)	
Tumor size (cm)	< 3.2	491 (58.6%)	285 (33.8%)	< 0.001	374 (44.7%)	402 (47.6%)	0.260	452 (55.1%)	324 (33.7%)	< 0.001
	≥ 3.2	347 (41.4%)	558 (66.2%)		462 (55.3%)	443 (52.4%)		369 (44.9%)	536 (62.3%)	
Tumor number	Single	570 (68%)	583 (69.2%)	0.686	565 (67.6%)	588 (69.6%)	0.401	574 (69.9%)	579 (67.3%)	0.270
	Multiple	268 (32%)	260 (30.8%)		271 (32.4%)	257 (30.4%)		247 (30.1%)	281 (32.7%)	
Vascular invasion	No	743 (88.7%)	625 (74.1%)	< 0.001	643 (79.9%)	725 (85.8%)	< 0.001	702 (85.5%)	666 (77.4%)	< 0.001
	Yes	95 (11.3%)	218 (25.9%)		193 (23.1%)	120 (14.2%)		119 (14.5%)	194 (22.6%)	
Extrahepatic metastases	No	813 (97%)	732 (86.8%)	< 0.001	758 (90.7%)	787 (93.1%)	0.073	778 (94.8%)	767 (89.2%)	< 0.001
	Yes	25 (3%)	111 (13.2%)		78 (9.3%)	58 (6.9%)		43 (5.2%)	93 (10.8%)	
Underlying cirrhosis	No	90 (10.7%)	160 (19%)	< 0.001	90 (10.8%)	160 (18.9%)	< 0.001	106 (12.9%)	144 (16.7%)	0.024
	Yes	748 (89.3%)	683 (81%)		746 (89.2%)	685 (81.1%)		715 (87.1%)	716 (83.3%)	
AFP (ng/mL)^a^	< 100	520 (62.1%)	464 (55%)	0.014	450 (53.8%)	534 (62.8%)	< 0.001	500 (60.9%)	484 (56.3%)	0.347
	≥ 100	271 (32.3%)	313 (37.1%)		321 (38.4%)	263 (31.1%)		282 (34.3%)	302 (35.1%)	
PIVKA-II (mAU/L)^b^	< 100	344 (41.1%)	230 (27.3%)	< 0.001	255 (30.5%)	319 (37.8%)	0.144	334 (40.7%)	240 (27.9%)	< 0.001
	≥ 100	205 (24.5%)	310 (36.8%)		252 (30.1%)	263 (31.1%)		222 (27%)	293 (34.1%)	

Abbreviations: *AFP* α-Fetoprotein; *HBV* Hepatitis B virus; *PIVKA-II* Protein induced by vitamin K absence or antagonist-II

^a^*n* = 1568, ^b^*n* = 1089

## Discussion

Hepatocellular carcinoma (HCC) is the one of the most common cancer and the fourth leading cause of cancer-related deaths worldwide [1]. Although HCC has highly aggressive behavior with poor prognosis, the relatively lower survival rate improved over the past decades [36]. Moreover, promising efficacy of new treatments have been introduced [37]. Therefore, there is a need to identify novel biomarkers for prognosis predictions and treatment strategies. The prognosis of patients with cancer relies not only on tumor-related factors, but also on host-related factors, including systemic immune statuses [38]. Various types of peripheral inflammatory cells have been found to provide promising prognostic survival value in patients with many cancers [19–23]*.* In this study, we evaluated the predictive value of peripheral inflammatory cells, and focus on individual cell types rather than combination scores. We found that pretreatment neutrophil, lymphocyte, and monocyte count was an independent prognostic factor for long-term OS in patients with HCC. By subgroup analyses, median neutrophil and monocyte counts increased with in BCLC stage. Conversely, lymphocytes tended to fall with advancing stages. These findings highlight the importance of the interaction between the host systemic immune cell activation and patient outcomes. In previous reports, the abundance of pretreatment peripheral inflammatory cells like neutrophils, lymphocytes, monocytes, and platelets has been reported to have promising prognostic values in predicting patient survival for several types of cancer. Generally, high baseline neutrophil, monocyte and platelet cell counts are associated with unfavorable prognosis, while higher pretreatment lymphocyte counts correlate with better survival [30, 32, 34, 39, 40]. Consistent with previous findings, our data showed that high neutrophils and monocytes and low lymphocytes are associated with reduced long-term OS in HCC.

Scientific evidence has associated infiltrating inflammatory cells with tumor initiation and progression [25, 41, 42]. Neutrophils, lymphocytes, and monocytes are critically involved in tumor progression in local tumor microenvironment and in peripheral blood [38]. Neutrophils are the most common white blood cell in the circulation and play an important roles in host defense, immune modulation, and tissue injury [43]. Neutrophils are considered as a one of the first immune cells enter the tumor microenvironment and interact with cancer cells, thereby playing an essential role in cancer progression [44].

In our study, neutrophil count was associated with tumor size, vascular invasion, extrahepatic metastasis, and AFP level, which suggest that increased neutrophil counts alter the tumor microenvironment and the formation of the inflammatory microenvironment thus promoting tumor growth and metastasis.

Besides neutrophils, monocytes are also of the myeloid lineage. Tumor-associated macrophages (TAMs) exist within the tumor microenvironment and are derived from circulating monocytes [45]. There is increasing evidence that TAMs are associated with cancer progression, and peripheral monocyte count has been reported as a useful prognostic marker [46–49]. Moreover, a prior study reported the relationship of these two factors, where peripheral monocyte count is associated with TAM density in the tumor. These researchers suggested that the underlying mechanism involves chemokines such as CCL2, which are produced by cancer cells and, promote the recruitment of peripheral monocytes to the tumor microenvironment [48].

Moreover, lymphocytes are usually recruited to the tumor microenvironment and engage in cell-mediated tumor responses. Numerous data have revealed that tumor infiltrating lymphocytes are associated with patient outcomes in various types of cancer [50–54], and lymphocyte phenotypes are fundamental for antitumor immunity and prognosis [38]. Therefore, reduced tumor infiltrating and peripheral lymphocytes might be indicative of impaired host antitumor response and favorable tumor microenvironments for cancer progression and dissemination. In concordance with this prediction, our data show that low peripheral lymphocyte counts correlates with vascular invasion reflecting their role in the tumor microenvironment.

There are many previous studies that peripheral inflammatory cell-based combination scores like NLR, LMR and PLR have a promising prognostic values in predicting patient survival for HCC in various treatment modalities [55–58]. In addition, several reports focusing on individual cell types to evaluated the prognostic value in predicting survival for HCC have been published, but these studies investigated patients who performed specific treatment [59, 60]. To our knowledge, until recently few studies have reported the prognostic value of pretreatment individual inflammatory cell alone in HCC regardless of treatment modalities. Recently, only one study has been reported focusing on neutrophil [40]. Therefore, this study can provide additive insight on the prognostic role of neutrophil as well as other individual inflammatory cell (lymphocyte, monocyte) as a relatively large scale and long-term follow up study.

There are several limitations associated with the present study. First, this study is a single-center, retrospective analysis with patient selection bias. Because we excluded patients who had active infection or inflammatory disease and those without data regarding clinical, laboratory, and imaging findings, patients who had advanced HCC with relatively high incidences of complications and missing data were excluded. However, our inclusion of individuals with near-complete patient data and long-term follow-up periods might in part compensate for this limitation. Second, the cut-off values of individual immune cells for patient stratification are arbitrary. Third, the mechanism of the correlation between individual cell counts and prognosis was not identified. In addition, we did not investigate how the interaction between immune cells contributes to tumor progression.

## Conclusion

We have found that pretreatment peripheral neutrophil, monocyte and lymphocyte counts are significant and independent prognostic predictors of long-term survival for patients with HCC. Considering the individual cells and their associations with tumor, neutrophils and monocytes play a role in promoting immune mediated tumor progression and lymphocytes have antitumor response roles. Due to their simplicity, reproducibility, and low cost, these immune cells provide a promising parameter for assessing HCC prognosis. The relationship between peripheral immune cells and the tumor microenvironment, as well as each immune cell’s phenotype, and function, interactions, and regulatory roles with other immune cells in the tumor have yet to be established. Further studies are needed to improve our understanding of the interaction between peripheral immune cells and the tumor microenvironment and validate the incorporation of this parameter into daily clinical practice. Furthermore, our results demonstrate that immune cells may provide clinically relevant therapeutic targets.

## Data Availability

The datasets used and/or analysed during this study are available from the corresponding author on reasonable request.

## Abbreviations

AFP: α-Fetoprotein
BCLC: Barcelona clinic liver cancer
HCC: Hepatocellular carcinoma
NLR: Neutrophil to lymphocyte ratio
LMR: Lymphocyte to monocyte
PLR: Platelet to lymphocyte ratio
OS: Overall survival
PIVKA-II: Protein induced by vitamin K absence or antagonist-II
